# Insect PRXamides: Evolutionary Divergence, Novelty, and Loss in a Conserved Neuropeptide System

**DOI:** 10.1093/jisesa/ieac079

**Published:** 2023-01-20

**Authors:** Sarah M Farris

**Affiliations:** Department of Biology, West Virginia University, 3139 Life Sciences Building, 53 Campus Drive, Morgantown, WV, USA

**Keywords:** diptera, pheromone biosynthesis activating protein, hugin, diapause hormone, capa

## Abstract

The PRXamide neuropeptides have been described in both protostome and deuterostome species, including all major groups of the Panarthropoda. Best studied are the insect PRXamides consisting of three genes: *pk/pban*, *capa*, and *eth*, each encoding multiple short peptides that are cleaved post-translationally. Comparisons of genome and transcriptome sequences reveal that while retaining its fundamental ancestral organization, the products of the *pk/pban* gene have undergone significant change in the insect Order Diptera. Basal dipteran *pk/pban* genes are much like those of other holometabolous insects, while more crown species have lost two peptide coding sequences including the otherwise ubiquitous pheromone biosynthesis activating neuropeptide (PBAN). In the genomic model species *Drosophila melanogaster*, one of the remaining peptides (hugin) plays a potentially novel role in feeding and locomotor regulation tied to circadian rhythms. Comparison of peptide coding sequences of *pk/pban* across the Diptera pinpoints the acquisition or loss of the hugin and PBAN peptide sequences respectively, and provides clues to associated changes in life history, physiology, and/or behavior. Interestingly, the neural circuitry underlying *pk/pban* function is highly conserved across the insects regardless of the composition of the *pk/pban* gene. The rapid evolution and diversification of the Diptera provide many instances of adaptive novelties from genes to behavior that can be placed in the context of emerging selective pressures at key points in their phylogeny; further study of changing functional roles of *pk/pban* may then be facilitated by the high-resolution genetic tools available *in Drosophila melanogaster*.

Neuropeptides (NPs) are short signaling peptides that are produced and released by neurons of the central nervous system, acting as juxtacrine, paracrine, and/or endocrine signals. NP genes can be recognized across taxa and share several key features (Jékely 2013, Mirabeau and Joly 2013, Elphick et al. 2018). At the synapse, NPs are packaged in dense core vesicles distinct from the clear vesicles containing small molecule transmitters; NPs can also be released from nonsynaptic areas of the cell membrane. NP genes encode an mRNA that is translated into a longer prepropeptide and subsequently enzymatically cleaved into multiple functional NPs with post-translational modifications (e.g., C-terminal amidation; Mains and Eipper 1999a). Recognition sites for peptide cleavage and post-translational modifications are often conserved across taxa as well. Nonetheless, functional NPs generated from the prepropeptide are often taxon-specific, and within species may vary according to cell type and developmental stage. Thus, a single NP gene may play multiple roles both within and between species. Additionally, NPs act through G-protein coupled receptors, and cross-reactivity of multiple related products of a single NP gene may occur (Mains and Eipper 1999b).

## Evolutionary History of Neuropeptides

Neuropeptide signaling is ubiquitous and highly conserved, with representatives of two metazoan NP gene families present even in single celled choanoflagellates, the likely sister group to the Metazoa. Cnidaria and Ctenophora have many NP genes in their genomes but most do not appear homologous to those of the Bilateria (Hayakawa et al. 2019); rather, most extant neuropeptide signaling systems have arisen, expanded, and diversified in the Bilateria (Li et al. 1999, Hewes and Taghert 2001, Burke et al. 2006, Conzelmann et al. 2013, Jékely 2013, Mirabeau and Joly 2013, Roch and Sherwood 2014, Elphick et al. 2018, Yañez-Guerra et al. 2022). Based on the widespread presence of NPs it is likely that as a group they serve fundamental core functions while at the same time remaining amenable to further adaptation and diversification. The availability of genome and transcriptome sequences for an increasing number of organisms allows comparisons of identified NP systems and reconstruction of changes in their makeup across taxa in relation to adaptations associated with species diversification. These analyses can thus provide a framework for understanding how even a highly conserved regulatory system has been differentially influenced by taxon-specific selection for life history, physiology, and behavior.

This paper begins as a review of the insect PRXamide neuropeptide system, followed by analysis of publicly available transcriptomes and genomes in one insect Order, the Diptera. Peptides in this family have been the subject of studies in diverse species such as cockroaches, locusts, moths, and flies; in particular, in the genomic model organism *Drosophila melanogaster* (Meigen, Diptera, Drosophilidae) PRXamide cells and circuits have been dissected in tremendous detail. Intriguingly, along with fundamentally conserved features of this system, *Drosophila* PRXamide structure and function illustrate some apparent novelties. Pairing this high-resolution template with comparative studies, especially in species carefully chosen for their place in the dipteran phylogeny and potentially key life history traits, will provide a sandbox for explorations of the nature of conservation, divergence, and evolutionary novelty in a framework encompassing adaptive modifications of genes, neurons, circuits, and behaviors.

## Structure of PRXamide Genes

The PRXamide NPs are ubiquitous across the protostomes (Jékely 2013, Mirabeau and Joly 2013; reviewed in Jurenka 2015, Elphick et al. 2018). In the Insecta where they are best characterized, PRXamide genes are highly conserved. Pheromone biosynthesis activating neuropeptide (PBAN) and 1–3 pyrokinins (PK), all characterized by an FXPRLamide C-terminus, are encoded on the *pk/pban* gene (termed *hugin* in Diptera, *pban* in Lepidoptera; Veenstra 2014). The pyrokinins (PKs) are confusingly referred to by several different monikers, including the subesophageal ganglion neuropeptides (α- β- and γ- SGNP) and hugin-γ/hugin (Sato et al. 1993, Bader, Wegener et al. 2007, Jurenka and Nusawardani 2011, Fodor et al. 2017). The *capa* gene encodes 1–2 periviscerokinins (referred to as CAPA-PVKs) that can be identified by a C-terminal sequence of FPRV/Iamide (Hull et al. 2021). Both *capa* and *pk/pban* genes also typically encode a tryptopyrokinin (trypto-PK) with a C-terminal sequence of WFGPRLamide; these peptides are designated diapause hormones DH1 and DH2, respectively (Jurenka 2015). Finally, ecdysis triggering hormone (ETH, usually encoded in two copies on the *eth* gene) is characterized by a longer consensus sequence, FFLKASKNVPRIamide or FFLKASKSVPRIamide (Melcher et al. 2006, Jurenka 2015). A regulator of ecdysis, the *eth* gene arose at the base of the Panarthropoda (de Oliveira et al. 2019). It is also not a true neuropeptide as it is produced by nonneuronal inka cells in the epitracheal gland (Žitňan et al. 1996); thus, the biology and evolution of *eth* will not be considered further here.

The deuterostome ortholog of insect PRXamides is *neuromedin U* (Predel and Nachman 2006, Lindemans et al. 2009, Jurenka 2015). In addition to conservation of neuropeptide genes, the G-protein coupled receptors (GPCRs) for PRXamides have coevolved with their ligands such that cross-phyla reactivity of ligands and receptors is possible. For example, the neuromedin U peptide is capable of binding and activating the PBAN GPCR (Choi et al. 2003).

## Evolutionary History of PRXamides in Arthropods

The evolutionary history of PRXamides in arthropods includes duplications across and within genes. A duplication of the phylogenetically oldest PRXamide gene (likely retained as the modern *eth* gene) gave rise to a gene with properties of both *pk/pban* and *capa*, termed *pk/capa* and containing both PK and PVK sequences (although in some species only PVKs have been identified; Neupert, Russell et al. 2009, Veenstra et al. 2012). A *pk/capa* gene has been identified in all of the major arthropod groups: the chelicerates, myriapods, and Pancrustacea (Derst et al. 2016). In species of tick (Acari) and Crustacea these neuropeptides are expressed in tissues including the nervous system (CNS) and feeding structures, and like the PRXamides of insects are capable of regulating muscle contraction (Torfs et al. 2001, Xiong et al. 2022). PKs also play neuromodulatory roles in the neural circuitry of the stomatogastric ganglion in the crustacean *Cancer borealis* (Stimpson, Decapoda, Cancridae) (Saideman et al. 2007, Dickinson et al. 2015, Yang et al. 2015).

A single gene encoding PKs, PVKs, and a newly acquired trypto-PK is observed in basal Hexapoda and in Xilbalbanus *tulumensis* (Yager, Nectiopoda, Speleonectidae), a species of the Remipedia and likely sister group to the Hexapoda (Derst et al. 2016, Diesner et al. 2021). Duplication of this gene into a PVK-encoding gene containing a trypto-PK (DH1) sequence (*capa*) and a separate PK-encoding gene (*pk/pban*; subsequently acquiring its own trypto-PK (DH2) sequence) occurred twice in basal Hexapoda, once in the Diplura (*Campodea augens* (Silvestri, Diplura, Campodeidae)) and in the apterygote insects belonging to the Zygentoma (*Thermobia domestica* (Packard, Zygentoma, Lepismatidae)). The latter is thought to represent the common ancestor possessing separate *capa* and *pk/pban* genes that are characteristic of extant insects. Interestingly, while species of the basal hexapod group Collembola possess a single gene, it produces two splice isoforms, one with PVKs and one with PKs. This separation of PVK and PK peptide production by one means or another appears to be ubiquitous in insects; as Derst et al suggest, early hexapods seem to have undergone strong selection for independent regulation of PRXamide gene products.

In most modern insect species, especially within the Holometabola, the makeup of the *capa* and *pk/pban* genes and their loci of expression and function are generally conserved. However, given the long evolutionary history and diversity of the insects, variation in the NPs encoded by these genes does exist. For example, large-scale rearrangements in peptide coding sequences coupled with gene duplications has been reported in species of two polyneopteran groups, the Dictyoptera and Orthoptera (Predel et al 2001, Redeker et al. 2017, Hao et al. 2019). The genome of the grasshopper *Locusta migratoria* (Orthoptera) contains three genes encoding multiple copies of trypto-PKs, two encoding several PKs including PBANs, and one *capa* gene encoding PVKs the trypto-PK DH1 (Redeker et al. 2017). Later, two more DH1 trypto-PK genes with multiple peptide sequences were isolated in additional species of Orthoptera and Blattodea (Hao et al. 2019). Grasshoppers also appear to have entirely lost coding sequences for the DH2 trypto-PK typically encoded on the *pk/pban* gene, leaving DH1 peptides solely responsible for inducing egg diapause. Loss of DH2 has also occurred independently within the Heteroptera and Neodiptera (Redeker et al. 2017, Ahn and Choi 2018, Hao et al. 2019). The number of non-PBAN PKs (α- β- and γ-SGNP is also variable: β- and γ-SGNP are found in many insect taxa, while α-SGNP only found in the sister groups Lepidoptera and Trichoptera; Apterygota, Heteroptera and some Homoptera. In contrast, the coding sequence for PBAN appears to be nearly ubiquitous across the Insecta, with the exception of the higher Diptera as demonstrated below.

## PRXamide-Expressing Neurons

Early studies using extracts of the subesophageal ganglion of the ventral nervous system (more recently termed the gnathal ganglion or GNG) identified this region of the central nervous system as a key source of signals inducing production of diapause eggs and pheromone biosynthesis. These signaling peptides were eventually identified as DH2 and PBAN, both encoded on the *pk/pba*n gene (Hasegawa 1964, Fukuda and Takeuchi 1967, Raina et al. 1989, Teal et al. 1989). Subsequent studies showed that the GNG contains *pk/pban* expressing cells of two types: interneurons whose axons are restricted to the CNS, and efferent neurons with axons projecting through peripheral nerves to neurohemal organs. The latter-produced peptides act as endocrine factors released into the hemolymph while the former act as neurotransmitters via synaptic or perisynaptic connections with other neurons (Teal et al. 1989). Efferent neurosecretory cell bodies are found as serially homologous pairs or bilateral clusters along the ventral midline of the GNG in each neuromere: the mandibular (Md), maxillary (Mx) and labial (Lb). Ablation studies in the silk moth *Bombyx mori* (Linnaeus, Lepidoptera, Bombycidae) showed that while these median neurosecretory cells all express the *pk/pban* gene, they do not process the prepropeptide into identical complements of mature peptides. Median neurosecretory cells of the mandibular and maxillary neuromeres (M_Md_ and M_Mx_) are required for initiation of pheromone biosynthesis, while M_Lb_ is necessary for inducing diapause (Ichikawa et al. 1996; using the nomenclature of Davis et al. 1996). This finding has been confirmed using antibodies specific to DH and PBAN peptides, demonstrating that while all express and produce the entire PBAN prepropeptide, *Bombyx* M_Md_ and M_Mx_ release mature PBAN peptide but not DH while M_Lb_ is responsible for release of DH but not PBAN (Ma et al. 2000, Hagino et al. 2010). Similarly, *Locusta migratoria* (Linnaeus, Orthoptera, Acrididae) M_Lb_ cells produce many trypto-PK peptides while PBAN and other PKs are restricted to M_Md_ and M_Mx_ (Redeker et al. 2017); differential peptide production has also been demonstrated in *Periplaneta americana* (Linnaeus, Dictyoptera, Blattidae) (Predel et al. 2007). Median neurosecretory neuron *capa* gene expression and peptide production can also be variable across species; in addition to expression of the *pk/pban* gene, the *capa* gene is also expressed in M_Lb_ in the moth *Manduca sexta* (Linnaeus, Lepidoptera, Sphingidae) (Loi and Tublitz 2004). Curiously, the M_Md_ and M_Mx_ usually share PRXamide gene expression/peptide production characteristics while the M_Lb_ differs; this is likely related to the morphology of these neurons’ projection in which M_Md_ and M_Mx_ are very similar while that of M_Lb_ targets some of the same tissues but via a unique trajectory as described below.

Additionally, PRXamides are nearly always produced and released by serially homologous neurosecretory neurons in the abdominal ganglia. These neurons are usually *capa-*expressing although rarely they express the *pk/pban* gene as well. PRXamide producing neurons can also be observed in thoracic ganglia and brain although this is more variable by species as well as developmental stage (Loi and Tublitz 2004, Predel and Wegener 2006).

Both GNG and abdominal median neurosecretory cells are ancient and can be observed with PRXamide immunostaining even in the basal apterygote *Lepisma saccharina* (Linnaeus, Zygentoma, Lepismatidae) and at least one nonhexapod member of the Pancrustacea (*Xibalbanus tulumensis,* Remipedia) (Diesner et al. 2021). Specifically, the GNG is an important locus of cells and circuits that regulate the animal’s physiology, life cycle, and autonomic functions somewhat analogous to the vertebrate brainstem (Hückesfeld et al. 2021). GNG median neurosecretory cells characteristically project to the retrocerebral complex in the head, specifically the corpora cardiaca (CC). In addition to releasing PRXamides onto the CC, these neurons also form dense secretory neurohemal networks on the surfaces of nerves and the GNG itself. The morphology of the abdominal neurosecretory neurons is also stereotyped, with axons leaving the ganglia via peripheral nerves to the perivisceral organs (PVOs) forming neurohemal surfaces on nerves along the way (Davis et al. 1996, Golubeva et al. 1997).

**Fig. 1.**
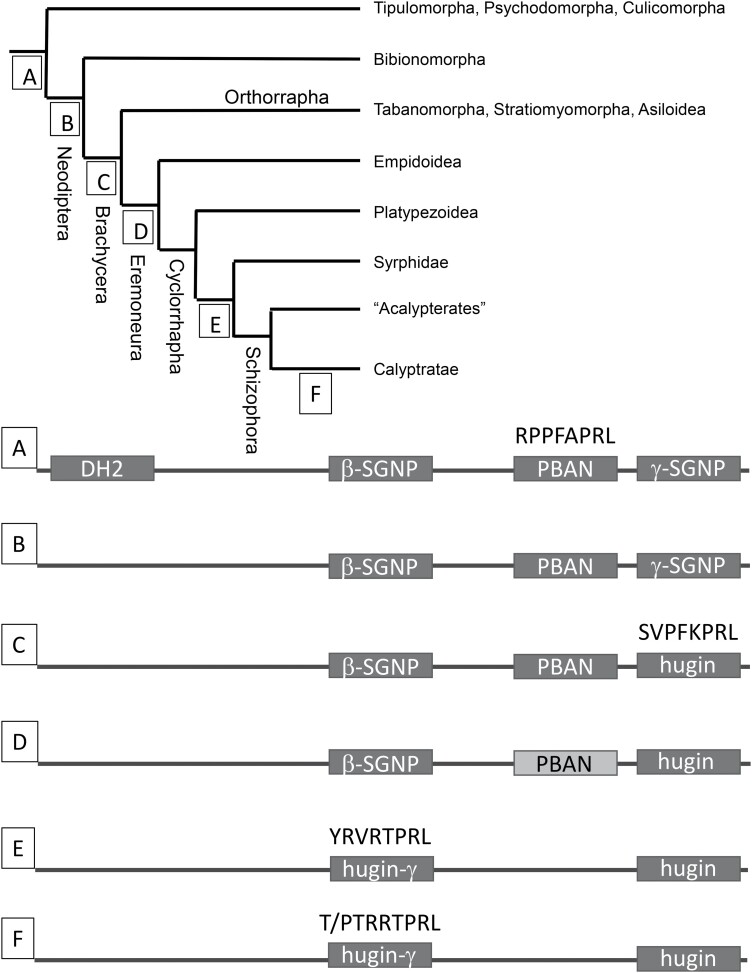
Changes in peptides coded by the *pk/pban/hugin* gene during the evolution of the Diptera. Letters A–F indicate correspondence of phylogenetic groups with mature pk/pban/hugin peptide sequences. DH2-diapause hormone 2; YRVRTPRL-hugin-γconsensus sequence in Syrphidae and acalypterate Schizophora; T/PTRRTPRL-hugin-γ consensus sequence in Calyptratae; SVPFKPRL-hugin consensus sequence in Brachycera; RPPFAPRL-PBAN consensus sequence in Tipulomorpha, Psychodomorpha, Culicomorpha, Bibionomorpha, and Orthorrapha. Lighter shaded box in the D gene sequence indicates PBAN-like peptide in Empidoidea and Platypezoidea that has lost the consensus sequence.

Anatomy of these efferent median neurosecretory neurons is particularly well-described in a number of moth species and can be used as a template for further comparisons (Fig. 1; Kingan et al. 1992, Davis et al. 1996, Golubeva et al. 1997). Anti-PBAN immunostaining and backfills of peripheral nerves reveal that median neurosecretory cells possess dorsally projecting neurites that travel contralaterally and produce bilateral arborizations in the dorsal GNG and anteriorly near the subesophageal foramen. M_Md_ and M_Mx_ cell axons leave the GNG via the maxillary nerve, enter the nervus corpora cardiaca V (NCCV) and end in the CC. They produce dense neurohemal networks along the way, forming a structure referred to as the corpus ventralis by (Golubeva et al. 1997). The precise anatomy and nomenclature of the cranial nerves utilized by the median neurosecretory cells vary by species, but the presence of M_Md_ and M_Mx_ efferent outputs to the CC appears relatively invariant across the insects (Tips et al. 1993, Bader, Colomb et al. 2007, Predel et al. 2007, Hellmich et al. 2014).

M_Lb_ efferent neurons have large, distinct soma with axons that ascend through the contralateral circumesophageal connective into the tritocerebrum and exit via the NCC-3 cranial nerve to the CC. The axons produce dense neurohemal terminals on the CC and then proceed to the corpora allata (CA) and dorsal aorta. Innervation of the CC by M_Lb_ appears highly conserved, although the additional innervation of the CA and aorta is not always reported. Interestingly, additional outputs of this neuron have been reported in some species. Most notably, Bräunig (1991) reports that in addition to targeting the CC, the M_Lb_ of the orthopteran *Locusta migratoria* produces ascending outputs that innervate the pharyngeal dilator muscles in the head while descending processes exit the abdominal ganglia and form neurohemal surfaces on nerves associated with the heart. Descending M_Lb_ processes to the VNC that exit via segmental nerves have also been observed in the cockroach (Predel et al. 2007), and innervation of the pharynx by M_Md_ and M_Mx_ but not M_Lb_ occurs in *Drosophila melanogaster* (Bader, colomb et al. 2007). Given the broad but patchy distribution of these neuron morphologies, it is possible that pharyngeal and descending efferent outputs of *pk/pban* expressing neurons are more common but underreported due to the small number of insects investigated to date, or that they are remnants of an ancestral innervation pattern, lost in many insects but retained in others, perhaps capable of reappearing in some taxa via a process such as generative homology where the developmental program is retained and can be redeployed and (Butler and Saidel 2000).

The second group of *pk/pban* expressing cells in the GNG are interneurons (INs) that provide processes to the brain, VNC, or both. In moths, INs that express the *pk/pban* gene are restricted to the maxillary neuromere (Blackburn et al. 1992, Kingan et al. 1992, Davis et al. 1996, Golubeva et al. 1997, Sun et al. 2005), however, INs are observed in the mandibular and/or labial neuromeres in other species (Hellmich et al. 2014). Together these neurons have a characteristic innervation pattern: descending processes travel along the lateral margin of the entire VNC, producing arbors along the way and ending with a broad arbor in the terminal abdominal ganglion. Ascending neurons arborize in the tritocerebrum and around the esophageal foramen, and travel dorsally through the pars intercerebralis (PI) and branch in an arc across the superior medial protocerebrum (Tips et al. 1993, Choi et al. 2001, Sun et al. 2005, Bader, Wegener et al. 2007, Predel et al. 2007, Choi et al. 2009, Ahn and Choi 2018, Hull et al. 2021).

## Order Diptera: PRXamides Under Rapid Diversification

The genomic model organism *Drosophila melanogaster* has an unusual modification to the *pk/pban* gene: loss of the PBAN coding sequence, which is present in all other insects investigated to date including basal Diptera. and acquisition of the hugin peptide that functions in feeding and circadian locomotor activity (Schoofs et al. 2014, Choi et al. 2015, Schwarz et al. 2021). The Diptera are a diverse and speciose insect Order that has undergone three rapid bursts of diversification: the first in the most basal clades (i.e., the former ‘Nematocera’), the second in the early Brachycera, and the third in the Schizophora, a group contained within the modern Cyclorrhapha. This last radiation occurred relatively recently in the Tertiary (around 65–40 mya). The Cyclorrhapha or ‘higher flies’ include *Drosophila melanogaster* and many other familiar species such as house flies and flesh flies. These insects are characterized by autapomorphies associated with molecular, cellular, and physiological processes. Students of developmental biology are well aware of the duplication and divergence of the *Hox3* gene into *zerknüllt* (*zen*), which is expressed in a novel embryonic tissue, the amnioserosa, and *bicoid* (*bcd*), one of the first axis patterning genes in the embryo (Schmidt-Ott 2000, Simpson 2002). Cyclorrhaphan maggot larvae are also unique in the loss of an external head and the formation of a puparium from the last instar exoskeleton rather than forming a pupal cuticle at metamorphosis. Additionally, the cyclorraphan subgroup Schizophora have a novel head structure, the ptilinum, that inflates with hemolymph to push through the puparium at adult emergence (Wiegmann and Yeates 2017, Bayless et al. 2021). As these examples illustrate, the Cyclorrhapha underwent a large-scale reorganization of developmental mechanisms during their radiation, and it would not be surprising if other seemingly fundamental processes were radically modified in comparison to other insects and more basal Diptera.

One way to investigate the relationship between adaptions and divergence of conserved cellular, molecular and physiological processes is to consult the wealth of publicly available genomes and transcriptomes available online. Using this approach, the *pk/pban* gene coding sequence was identified in 155 dipteran species, and alignment of putative mature neuropeptides was performed to assess conservation and divergence of consensus sequences. This data was then mapped onto a published phylogeny of Diptera and key locations of change in the *pk/pban* coding sequence identified.

## Materials and Methods

The phylogeny of Diptera published by (Wiegmann et al. 2011) was used to devise a strategy for genome, transcriptome, and protein sequence searches so that the location of *pk/pban* alterations could be pinpointed to distinct clades (e.g., Neodiptera, Orthorrhapha etc.). The website InsectBase 2.0 (http://v2.insect-genome.com/; Yang et al. 2022) was used as a starting point for searches by gene name (*pban* or *hugin*) for protein sequences from major taxonomic groups of Diptera. These protein sequences were used to search the National Center for Biotechnology Information (NCBI) Nucleotide and Sequence Read Archive (SRA) databases at the National Center for Biotechnology Information website (Sayers et al. 2022) using the tblastn search tool (Altschul et al.1990). Retrieved sequences were aligned using the Clustal Omega web tool (Sievers et al. 2011). The WebLogo tool was used for visualization of peptide consensus sequences (Schneider and Stephens 1990, Crooks et al. 2004). Mature peptide sequences were identified using criteria employed in previous studies of the dipteran *pk/pban* gene (Bader, Wegener et al. 2007, Choi et al. 2015): the C-terminal sequence (PRXGK/R) specifically characteristic of the pyrokinins, and N-terminal cleavage sites consisting of mono- or dibasic residues (K, R).

## Results

**Fig. 2.**
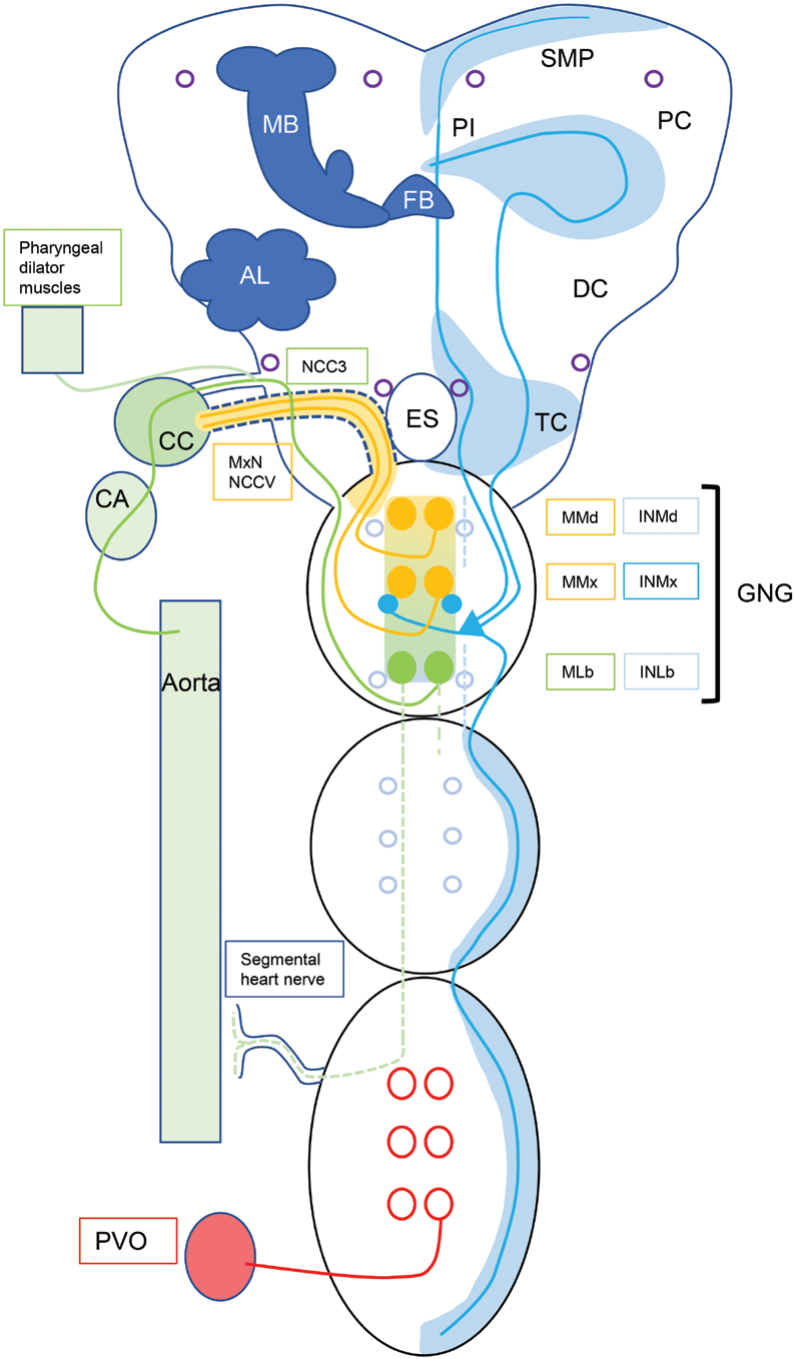
Groundplan of *pk/pban/hugin* expressing neurons and their peripheral targets. Light blue open circles and blue/green dotted lines – neurons and processes in the GNG and VNC that are observed in some but not all species. Purple open circles – neurons observed in the brain of some but not all species. Brain structures: MB-mushroom bodies, FB-fan-shaped body, AL-antennal lobe, SMP-superior median protocerebrum, DC-deutocerebrum TC-tritocerebrum, ES-esophageal foramen. GNG-gnathal ganglion: MMd, MMx, MLb-median mandibular, maxillary, and labial efferent neurons. INMd, INMx, and INLb-mandibular, maxillary, and labial interneurons. Nerves: NCC3-nervus corpora cardiaca 3, NCCV-nervus corpora cardiaca 5, MxN-maxillary nerve. Peripheral targets: CC-corpora cardiaca, CA-corpora allata, PVO-perivisceral organ.

Early studies posited that the primary functional peptide sequence in the *Drosophila pk/pban* gene (called the *hugin* gene in this species) is derived from the ancestral PBAN sequence. Comparisons of *pk/pban* coding sequences gleaned from publicly available genomes and transcriptomes across the Diptera instead support a scenario in which the PBAN peptide was lost entirely and the neighboring γ-SGNP sequence diverged into the hugin consensus sequence (SVPFKPRL) at the base of the Neodiptera (C, Fig. 2; Supplementary Data 1 [online only]). As observed for most insects the PBAN sequence is present in basal Diptera and resides between β-SGNP and γ-SGNP. It has a conserved consensus C-terminal sequence (RPPFAPRL; C, Fig. 2). Beginning with the Eremoneura, the PBAN consensus sequence begins to diverge but retains the PRXamide motif (D, Fig. 2; Supp. Data 1 [online only]). Complete loss of the PBAN consensus sequence including the PRXamide motif is found to have occurred in the ancestor of the Syrphidae and Schizophora (E, Fig. 2). At the same time, the β-SGNP sequence acquired the conserved γ-hugin consensus sequence for this group (YRVRTPRL) later modified to a T/PTRRTPRL consensus sequence characteristic of the calypterates (Kean et al. 2002, Bader, Wegener et al. 2007). The hugin sequence is still preceded at its N-terminus by the dibasic cleavage site (KK) that in more basal Diptera follows the amidation target glycine (G) of the PBAN sequence. The hugin peptide also contains 8 amino acids including the PRX motif which is more typical of γ-SGNP than of PBAN, the latter of which is characteristically around 30 amino acids long (Jurenka 2015).

Previous authors have introduced several lines of evidence suggesting that γ-hugin (residing in the N-terminal direction from PBAN and termed β-SGNP in most insects) does not produce a functional mature peptide in *Drosophila* (Predel and Nachman 2006, Choi et al. 2015); however, the PRXamide motif and C-terminal cleavage and amidation sites as well as the C-terminal consensus sequences are conserved, suggesting that this sequence may still play an adaptive role in these insects (Fig. 2).

The hugin peptide sequence of Orthorrapha and Eremoneura is also highly conserved (SVPFKPRL) although single amino acid substitutions characterize some taxa (e.g., SVQFKPRL in the higher Calyptratae). In *Drosophila melanogaster* the hugin peptide plays a potentially novel role in regulation of circadian locomotor rhythms and feeding behaviors (Sun et al. 2014, Schoofs et al. 2014, King et al. 2017, Schwarz et al. 2021). Given the conserved sequence motif of the hugin peptide it is likely that these functions are supported by the hugin peptide throughout the Brachycera. Interestingly, the vertebrate ortholog of *pk/pban* and *hugin*, *neuromedin U*, has also been shown to regulate feeding in rodents (Martinez and O’Driscoll 2015), perhaps suggesting that his NP system is amenable to incorporation into regulatory pathways for control of feeding behaviors.

## Potential for Novel Functions of Dipteran PRXamides

Multiple, sometimes overlapping functions of the peptide products of the *pk/pban* and *capa* genes have been identified in a diverse array of insect species. Early studies of the physiology of visceral muscle contraction in species of Dictyoptera and Orthoptera insects identified several classed of neuropeptides. Those referred to as myotropins, locustapyrokinins, or leucopyrokinins were later shown to be encoded by the *pk/pban* gene and were subsequently isolated in other species (Tips et al. 1993, Predel et al. 2001, Yaginuma and Niimi 2016). PBAN was identified first in Lepidoptera as the signal initiating pheromone biosynthesis by the pheromone glands, a finding that has since been replicated in species outside of the Lepidoptera (Kitamura et al. 1989, Raina et al. 1989, Tillman et al. 1999, Choi and Vander Meer 2012). The peptide MRCH (melanization and reddish coloration hormone) was identified in the moth *Spodoptera litura* (Fabricius, Lepidoptera, Noctuidae) as a regulator of environmentally-controlled larval cuticle pigmentation and later shown to correspond to the PBAN neuropeptide of *Bombyx mori* (Matsumoto et al. 1990). Similarly, in brachyceran Diptera, pyrokinins decrease the time needed for puparium formation and cuticle tanning (Zdarek et al. 1997). These studies hint at a general role in cuticle physiology during development, although it should be noted that in these and other physiological and immunocytochemical studies the exact identity of the PK, and even the difference between PKs and PVKs, was difficult to determine due to the sequence similarities among the peptides.

The trypto-PKs DH1 and DH2 encoded by the *capa* and *pk/pban* genes act on the ovaries of adult female Lepidoptera and are transferred to developing oocytes, where they regulate production of eggs that will begin diapause after fertilization (Yamashita 1996, Hao et al. 2019). In species in which diapause occurs in the pupa rather than the egg, DH2 appears to initiate the end of diapause rather than the beginning (Xu and Denlinger 2003). Periviscerokinin was initially isolated as a facilitator of visceral muscle contraction in *Periplaneta americana*, as a cardioaccelatory peptide in *Manduca sexta* (CAP2b), and as diuretic or anti-diuretic hormones in several insect species.

Insect genomes contain four GPCRs whose products have different binding affinities to each peptide encoded by the *pk/pban* and *capa* genes. More than one peptide typically can bind each receptor (reviewed by Jurenka 2015); although the functions of the additional 2–3 pyrokinins encoded by the *pk/pban* gene are poorly understood, their ability to bind these GPCRs suggests they may participate in some aspect of the functions already described for the better-known peptides (Ma et al. 1996, Kean et al. 2002, Choi et al. 2003). In *Drosophila melanogaster*, binding assays reveal that both hugin and PBAN peptides bind with high affinity to PK GPCRs (Choi et al. 2003), suggesting that the loss of the PBAN peptide and gain of hugin functionality need not require dramatic changes in structure and function of the cognate receptor. Rather, a newly acquired peptide such as hugin may tie ancestral functions of pyrokinins to a novel function, as discussed below.

## Hugin Peptides and Neurons in Diptera

A closer look at *hugin* expressing neurons afforded by the genetic tools available in *Drosophila melanogaster* confirms the conserved nature of the *pk/pban* neurosecretory circuits and uncovers high-resolution details of cell structure and function. Early studies using a polyclonal antibody against the C-terminus of PBAN detected hugin protein in larval ventral midline cells in each neuromere of the GNG and in abdominal ganglia (Choi et al. 2001). The abdominal neurons project to the perivisceral organs in the periphery, while those of the GNG have processes in the protocerebrum and tritocerebrum of the brain, the ventral nerve cord, and corpora cardiaca of the retrocerebral complex (termed the ring gland (RG) in Diptera). A similar pattern was observed in adults, with the addition of a pair of immunopositive neurons in the protocerebrum and a small group of neurons in the tritocerebrum. A closer look at individual *hugin*-expressing neurons in the mandibular and maxillary neuromeres of the GNG reveals that each neuron innervates one of four targets: efferent neurons project to the RG or pharyngeal dilator muscles (PH), and INs target the superior medial protocerebrum (PC) or have descending axons (VNC). All have fine processes in the dorsal GNG and TC around the esophageal foramen (Bader, colomb et al. 2007, Bader, Wegener et al. 2007; Mizuno et al. 2021).

An interesting and potentially novel feature of *Drosophila* GNG neurosecretory neurons is the loss of *hugin* gene expression in M_Lb_ and redeployment of these neurons in a new circuit in which they instead express *capa*. Single cell neuroanatomical reconstructions identify efferent neurons innervating the RG and PH that appear to reside only in the mandibular and maxillary neuromeres. (Melcher and Pankratz 2005, Hückesfeld et al. 2016). A pair of large ventromedial neurons reside in the labial neuromere that express *capa* but not *hugin* (Predel and Wegener 2006). As described above, M_Lb_ frequently has a different peptidergic content from the M_Mx_ and M_Md_ cells, including expression of both *capa* and *pban* in the moth *Manduca sexta* (Loi and Tublitz 2004, Neupert, Huetteroth et al. 2009). Despite co-expression of both PRXamide genes the lepidopteran M_Lb_ has the ‘typical’ morphology of projections through NCCIII to the CC, CA and aorta (Ichikawa et al. 1995, Davis et al. 1996, Golubeva et al. 1997). The same is true for basal dipteran mosquitos, where M_Lb_ co-expresses *capa* and *pk/pban* and efferents from all three GNG neuromeres project to the ring gland (Hellmich et al. 2014). Drosophila *capa*-expressing M_Lb_ have processes in the ring gland that are reported to provide dendrites to adipokinetic hormone (AKH) cells in the CC. Additionally, they have a potentially novel efferent output onto the foregut proventriculus (Kean et al. 2002, Melcher and Pankratz 2005, Koyama et al. 2021). CAPA-PVKs are well established diuretic or anti-diuretic factors that typically act on the Malphigian tubules or the gut but a direct projection from the GNG to the gut has not been reported in other insects (Sajadi et al. 2020, Zandawala et al. 2021).

A detailed connectome of hugin-producing neurons is being constructed in *Drosophila* as well. Interneurons are characterized by mixed pre- and postsynaptic processes, and all neurons receive input in the GNG. One source of GNG inputs is Gr66a gustatory afferents from the pharynx, external head and abdomen that are specifically tuned to detect bitter, aversive substances (Melcher and Pankratz 2005, Bader, Wegener et al. 2007, Schlegel et al. 2016). Additionally, hugin neurons receive input from descending PI neurons in the dendritic field of the ventrolateral esophageal foramen (Schlegel et al., 2016, King et al. 2017). The PC hugin neurons form a complex circuitry in the medial and superior protocerebrum, with reciprocal connections with the median neurosecretory cells of the PI and inputs from neurons that also target the PI. Finally, VNC neurons are both post- and presynaptic within the VNC suggesting a neuromodulatory role that is sensitive to local feedback (Miroschnikow et al. 2018).

## Functions of *Drosophila* Hugin

Initial studies of *Drosophila hugin* function focused on regulation of feeding as manipulation of GNG hugin neuron activity can disrupt or initiate feeding behaviors (Melcher and Pankratz 2005, Melcher et al. 2007, Sun et al. 2014, Hückesfeld et al. 2016). This function appears to be especially linked to aversive gustatory cues, supported by the inputs to GNG hugin neurons from aversive *Gr66a*-expressing gustatory neurons as described previously. Further investigations began to untangle roles of specific GNG hugin neurons: locomotor speed is specifically regulated by the VNC projecting hugin neurons while the remaining neurons are required for suppression of feeding and onset of locomotion (Schoofs et al. 2014). A potential adaptive scenario for hugin in this role is revealed by the necessity of hugin expressing neurons for larval evasion when confronted with bacterial pathogen-infected food (Surendran et al. 2017).

Recently, a novel population of hugin producing interneurons (Hugin^TS^ in the third thoracic segment) was found to respond to rising glucose levels by inhibiting diuretic hormone 44 (DH44) producing neurons and promoting cessation of feeding behavior (Oh et al. 2021). Median neurosecretory cells in the PI produce (DH44) and send descending processes to the ventrolateral esophageal foramen neuropil where they overlap with the dendrites of GNG hugin neurons expressing the DH44 receptor (McKellar 2016, King et al. 2017, Schwarz et al. 2021). Circadian release of DH44 is an important regulator of neurons that control sleep: activity rhythms suggesting that hugin neurons also contribute to daily behavioral rhythms (Cavanaugh et al. 2014). GNG hugin neurons with axons in the VNC are presynaptic to motor neurons providing a means for contributing to circadian regulation of locomotor patterns (King et al. 2017). The modulation of motor output by pyrokinins is well-documented in systems such as the stomatogastric ganglion of the crab *Cancer borealis*, potentially suggesting conservation of this particular role (Saideman et al. 2007). *Drosophila* hugin efferents projecting to the CA of the ring gland also express the receptor for DH44 and thus are likely to participate in circadian functions of this neuroendocrine gland (Mizuno et al. 2021). Finally, GNG hugin neurons decrease activity and suppress circadian activity under sleep deprivation conditions, possibly via direct connections between ascending hugin interneurons and sleep homeostasis regulating neurons in the protocerebrum (Schwarz et al. 2021).

## Evolutionary Significance of Divergence of PK/PBAN Peptides

The *pk/pban* gene of Diptera has retained its ancestral structure but has also undergone significant evolutionary divergence during the evolution of the Diptera. How have the higher flies compensated for the lack of PBAN, and how did the novel functions of hugin arise? The PBAN peptide is highly conserved across the entire Insecta and plays an equally conserved role in the regulation of pheromone biosynthesis (reviewed by Jurenka 2015). In some moth species, PBAN release and subsequent pheromone biosynthesis occur on a circadian basis (reviewed by Groot 2014, Iglesias et al. 1999, Tawata and Ichikawa 2001, Závodská et al. 2009). This suggests that the *pk/pban* expressing neural circuitry in the GNG is ancestrally linked to circadian networks in the bran, likely facilitating the incorporation of the newly acquired hugin peptide into this regulatory network as observed in *Drosophila*. The adaptive significance of the novel function of hugin in the higher Diptera remains to be determined, and a search for similar changes in the *pk/pban* gene in other insects should provide insight.

Loss of the PBAN coding sequence in the ancestor of Syrphidae and Schizophora is especially curious, as pheromones play an important role in fly behaviors including attraction of the opposite sex. What aspects of pheromone physiology biology differ in the higher flies when compared with other insects? An intriguing study in *Drosophila* demonstrates that circadian pheromone biosynthesis in the absence of the PBAN peptide is instead directly regulated by release of pigment-dispersing factor (pdf) from circadian rhythm generating clock neurons in the brain (Krupp et al. 2013). Loss of the PBAN sequence in the ancestor of Syrphidae + Schizophora could be explained by the loss of its adaptive function as a regulator of pheromone biosynthesis following recruitment of the pdf peptide for this role.

Another factor that may have influenced the regression and loss of the PBAN peptide is the tissue responsible for pheromone production. In the higher flies, pheromone biosynthesis occurs in large epidermal secretory cells called oenocytes while basal Diptera possess PBAN-responsive abdominal pheromone glands like those observed in most other insects (Blomquist et al. 1987, Spiegel et al. 2016). Perhaps oenocytes are more amenable to direct regulation by the circadian clock, contributing to the obsolescence of the PBAN peptide in higher Diptera.

Finally, pheromone biosynthesis by some schizophoran species appears to be uniquely regulated by ecdysteroid hormones, as shown by studies of *Drosophila melanogaster* and *Musca domestica* (Linnaeus, Diptera, Muscidae). In contrast, juvenile hormone is more typically employed as a regulator of pheromone biosynthesis, (Blomquist et al. 1987, Tillman et al. 1999). This rather dramatic change in endocrine regulatory pathways may also have contributed to the loss of the role of PBAN.

The Order Diptera is ideal for continued studies of neuropeptide evolution. First, the genetic model species *Drosophila melanogaster* provides a means for high resolution dissection of genetic, cellular, and physiological processes. Second, the diversity of species can be employed in comparative studies to identify the adaptive significance of divergence of the PBAN gene in terms of life history, ecology, and behavior. This approach has already been used to trace the many genetic and developmental novelties of *Drosophila* to their evolutionary origins (e.g., the origin of the *Drosophila* anterior determining gene *bicoid* as reviewed by McGregor 2005). Further novelties in neuropeptide systems are likely present and waiting to be studied. Utilizing the wealth of genomes and transcriptomes now available, changes in gene sequence may be mapped not only onto phylogeny, but onto nervous system function, ecology, and behavior. Once such a framework is in place, emerging technologies such as CRISPR may be used to gain a more detailed mechanistic understanding of cellular and molecular systems to species in addition to *Drosophila*.

## Author Contribution

SMF Conceptualization; Data curation; Formal analysis; Investigation; Methodology; Visualization; Writing – original draft; Writing – review and editing.

ieac079_suppl_Supplementary_Data
